# Virtual Screening of Small Molecules Targeting BCL2 with Machine Learning, Molecular Docking, and MD Simulation

**DOI:** 10.3390/biom14050544

**Published:** 2024-05-01

**Authors:** Abtin Tondar, Sergio Sánchez-Herrero, Asim Kumar Bepari, Amir Bahmani, Laura Calvet Liñán, David Hervás-Marín

**Affiliations:** 1 Department of Computer Science, Multimedia and Telecommunication, Universitat Oberta de Catalunya (UOC), 08018 Barcelona, Spain; ssanchezherre@uoc.edu; 2 Stanford Deep Data Research Center, Department of Genetics, Stanford University, Stanford, CA 94305, USA; abahman@stanford.edu; 3 Department of Pharmaceutical Sciences, North South University (NSU), Dhaka 1229, Bangladesh; asim.bepari@northsouth.edu; 4 Telecommunications and Systems Engineering Department, Universitat Autònoma de Barcelona (UAB), Carrer Emprius, 2, 08202 Sabadell, Spain; laura.calvet.linan@uab.cat; 5 Department of Applied Statistics, Operational Research, and Quality, Universitat Politècnica de València (UPV), 03801 Alcoy, Spain; daherma@upv.edu.es

**Keywords:** small molecules, virtual screening, BCL2, cancer therapeutics

## Abstract

This study aimed to identify potential BCL-2 small molecule inhibitors using deep neural networks (DNN) and random forest (RF), algorithms as well as molecular docking and molecular dynamics (MD) simulations to screen a library of small molecules. The RF model classified 61% (2355/3867) of molecules as ‘Active’. Further analysis through molecular docking with Vina identified CHEMBL3940231, CHEMBL3938023, and CHEMBL3947358 as top-scored small molecules with docking scores of −11, −10.9, and 10.8 kcal/mol, respectively. MD simulations validated these compounds’ stability and binding affinity to the BCL2 protein.

## 1. Introduction

The BCL2 (B-cell lymphoma 2) family of proteins is essential in arbitrating mitochondrial outer membrane permeabilization (MOMP). It plays a pivotal role in regulating apoptosis, a form of programmed cell death crucial for maintaining cellular homeostasis [1]. The dysregulation of the BCL2 family of proteins is often implicated in cancer, making the BCL2 family a significant target for cancer therapy [2].

Anti-apoptotic BCL2 family members, which include BCL2 (Apoptosis regulator Bcl-2), BCL-XL (B-cell Lymphoma-Extra-Large), BCL2L2 (Bcl-2-Like Protein 2 or Bcl-W), MCL-1 (Induced Myeloid Leukemia Cell Differentiation Protein Mcl-1), and (BCL2-Related Protein A1), share a structural framework that comprises four conserved BCL2 homology (BH) domains (BH1-4) [3]. BCL2 is the prototype of the family. BCL2’s anti-apoptotic activity is associated with the integrity of its BH domains. BCL-XL is very similar in structure to BCL2 [4]. BCL2L2 is also structurally similar to BCL2 and BCL-XL. MCL-1 possesses a unique binding site for certain BH3-only proteins, making it a distinct target for small molecule inhibitors [5]. CL2A1 has a short half-life and exhibits tissue-specific expression [6]. 

Pro-apoptotic family members such as BAX (Apoptosis regulator BAX or Bcl-2-like protein 4 (BCL2-L-4)), BAK (Bcl-2-antagonist/killer 1), and BOk (Bcl-2-related ovarian killer protein) are often referred to as the effectors of apoptosis [7]. BAX normally resides in the cytosol inactive and translocates to the mitochondria upon apoptotic stimuli [8]. BAK is constitutively integrated into the mitochondrial outer membrane and similarly undergoes conformational changes during apoptosis, oligomerizing to form pores (BAK1 BCL2 Antagonist/Killer 1 (*BAK1 BCL2 Antagonist/Killer 1 (Homo Sapiens (Human)*) n.d.), (Uniprot, n.d.). BOK is believed to function similarly [9,10].

The impacts of anti and pro-apoptotic proteins on cancer have been extensively studied. For example, Kunac et al. explored the expression of apoptosis regulators Bcl-2 and Bax in colorectal carcinoma, finding variable patterns between tumor grades and stromal cells. Their findings suggest that Bcl-2 is more expressed in the lamina propria of low-grade cancers. At the same time, Bax is more in the epithelium, with these patterns potentially serving as prognostic markers and influencing treatment approaches for colorectal cancer [11]. Kawiak and Kostecka examined the regulation of BCL2 family proteins in estrogen receptor-positive breast cancer, revealing that anti-apoptotic BCL2 proteins are often overexpressed, contributing to resistance to endocrine therapy [12]. 

In recent years, there has been an exponential increase in using computational methods for drug discovery, such as virtual screening. Traditional virtual screening methods rely on molecular docking, which predicts the preferred orientation of one molecule to a second when bound to each other to form a stable complex [13]. While molecular docking has its merits, it often inaccurately assumes molecules to be rigid. This simplification overlooks the inherent flexibility of proteins and biological molecules, which dynamically change conformations due to environmental interactions and are critical for their functions. Consequently, docking may miss viable drug candidates or predict unfeasible interactions within the cell’s dynamic milieu. Furthermore, it fails to fully incorporate the complex interplay of molecular interactions and environmental conditions—such as solvent effects, pH variations, and the presence of ions or other molecules—that influence the stability and specificity of protein–ligand complexes. These oversights limit the precision and effectiveness of virtual screening in pinpointing potential drugs, underscoring the need for more advanced computational methods that better reflect biological complexities [14]. 

Several studies have used virtual screening to discover small molecules for treating cancer. For example, Valentini et al. utilized machine learning for virtual screening to discover new molecules, IS20 and IS21, targeting BCL2 family proteins. These small molecules showed potential in cancer treatment by promoting apoptosis in various cancer cell lines [15]. Zhou et al. used a machine learning method to identify a novel small molecule, DC-B01, that showed high binding affinity to the BCL-2 BH4 domain, disrupting its function and inducing apoptosis in cancer cells [16]. 

Deep neural networks (DNNs) present an advanced alternative to traditional methods. They are a subset of machine learning techniques that can learn from and make data-based decisions [17]. In drug discovery, DNNs have showcased their potential to predict drug–protein interactions efficiently, reduce the time required for drug development, and offer more precise predictions than some conventional methods [18]. Their architecture, which allows them to capture non-linear relationships, makes them especially suitable for modeling biological systems, which are inherently complex [19]. 

Chen et al. utilized a deep learning neural network to predict the inhibitory potential of small molecules on the MMP13 enzyme, which is crucial for cancer cell tumor development. Their study achieved notable R-squared values on both training and test datasets, highlighting the promise of AI in advancing cancer treatment discovery [20]. Another study by Zhang and colleagues highlighted the ability of DNNs to predict BCL2 inhibitors with high accuracy and efficiency, emphasizing the technique’s potential to speed up the drug discovery process [21]. 

Similarly, RF algorithms have become integral in drug discovery, offering robust predictive models that guide researchers in identifying novel therapeutic compounds [22,23,24]. Random forest has been used in several studies on the BCL2 protein family. For instance, Ko et al.’s research on 635 stage I non-small cell lung cancers (NSCLC) revealed a synergistic effect of cyclin A2 overexpression and negative Bcl-2 expression on worsening recurrence-free survival (RFS) [25]. Derenzini et al.’s research successfully integrated gene expression profiling with clinical prognostication in diffuse large B-cell lymphoma (DLBCL), particularly highlighting the role of MYC and BCL2 in this type of cancer [26]. Urban et al. presented a comprehensive approach to CLL treatment by evaluating the prognostic value of BCL2 and BTK activity at the outset of therapy [27]. Utilizing a random forest (RF) classifier for high-resolution immune profiling, the study successfully predicted MRD status post-treatment, emphasizing the significance of BCL2 and RF analysis in determining treatment outcomes. This result highlights the potential of these biomarkers in developing personalized, precision therapeutic strategies for CLL patients.

The use of random forest (RF) instead of deep neural networks (DNN) for virtual screening in the discovery of cancer drug therapeutics offers several potential advantages. Firstly, RF algorithms are typically faster to train than DNNs, a crucial factor when screening large databases of small molecules [28]. This feature is particularly beneficial in early-stage drug discovery, where quick iteration is critical. Additionally, RF models often require fewer data to achieve reliable performance, an advantage in scenarios where data on new compounds are limited. Unlike DNNs, RF provides better interpretability, allowing researchers to understand which features of the molecules are most influential in their activity, thereby guiding further modifications [29]. 

Moreover, RF is less prone to overfitting compared to DNNs, especially in cases with smaller datasets, making it a more robust choice for screening diverse chemical spaces [30]. Finally, RF’s ability to handle imbalanced datasets is crucial in drug discovery, where active compounds are often rare [31]. While DNNs can offer deeper insights with sufficient data and computational resources, RF’s simplicity, speed, and interpretability make it a compelling choice in the initial phases of cancer drug therapeutics discovery [32]. 

Our study presents a novel methodology to bridge these gaps, employing an integrated approach that combines RF, molecular docking, and MD simulations. This method enhances the prediction accuracy for potential BCL-2 inhibitors, leveraging RF for screening and molecular docking with MD simulations for detailed analysis of binding affinity and stability. Moreover, our distinctive ligand library, sourced from RCSB PDB and enriched with unique molecules from ChEMBL, DrugBank, and ZINC15, enables a comprehensive exploration of chemical space. This study aimed at addressing the previous screening limitations and improving the likelihood of discovering novel, effective BCL2 inhibitors, marking a significant step forward in cancer therapy research.

## 2. Methods

### 2.1. Activity Dataset

We first downloaded the PubChem [33] bioassay data for BCL2 from https://pubchem.ncbi.nlm.nih.gov/gene/596#section=BioAssays, accessed on 22 March 2024. The data were filtered to keep records where the assay type (aidtype) was ‘Confirmatory’. We then retrieved chemical data for each assay ID (AID) where the ‘PUBCHEM_ACTIVITY_OUTCOME’ was ‘Activity’. Duplicates and missing records for ‘PUBCHEM_CID’ were removed, and Lipinski descriptors were generated using RDKit (“RDKit: Open-source cheminformatics. https://www.rdkit.org, accessed on 22 March 2024”, 2023) and saved as a CSV file. Next, we split the data (stratified for ‘Activity’) and reserved 5% of records (194 compounds) for external validation; the remaining 95% were used for training and testing the machine learning models. The PubChem fingerprints were generated from SMILES using PaDEL-Descriptor [34], automating the process with the python wrapper padelpy (https://github.com/ecrl/padelpy, accessed on 22 March 2024). 

### 2.2. Ligand Library for Screening

To prepare the ligand library for screening, we retrieved bound ligands from the 3D structures of the human apoptosis regulator Bcl-2 protein (BCL2) (https://www.uniprot.org/uniprotkb/P10415, accessed on 22 March 2024) from the RCSB PDB database (https://www.rcsb.org/, accessed on 22 March 2024).

For each ligand, we performed ligand-based screening from the SwissSimilarity webserver [35] and merged the data to create the dataset for screening BCL2 inhibitors. The PubChem fingerprints and Lipinski descriptors were generated as described above.

### 2.3. Deep Neural Network (DNN) Machine Learning Models

The target variable was ‘Activity’ with binary values ‘Inactive’ and ‘Active’. The PubChem fingerprints were treated as the features. We split the data into a training set (80%) and a validation set (20%), stratified for ‘Activity’. The model started with an input layer with 881 neurons and a rectified linear unit (ReLU) activation function. In contrast, the output layer consisted of a single neuron with a sigmoid activation function. 

We performed tuning with a grid search for hyperparameters to optimize the number of hidden layers (either two or three) and the number of neurons in each hidden layer [(512, 256, 128), (256, 128, 64), (128, 128, 64), (512, 256), (256, 128), (128, 64)]. The number of training epochs was 50 or 100, and the batch size was 32 or 64. The model was configured using the adaptive moment estimation (Adam) optimization algorithm with the ‘binary_crossentropy’ as the loss function. The ‘ROC-AUC’ metric was employed to assess the model’s performance during training and validation. The model was trained with a callback to save the model based on validation loss.

For model generation and validation, we used the Python libraries Sklearn [36] and TensorFlow [37], with dependencies in a Jupyter Notebook [38].

### 2.4. Random Forest (RF) Machine Learning Model

We generated an RF binary classification model with Sklearn [36]. The target variable ‘Activity’ labels were mapped to binary values, ‘Inactive’ as 0 and ‘Active’ as 1. The train_test_split function of Sklearn divided the data into training and testing subsets. We performed hyperparameter tuning using a grid search approach for the number of estimators [100, 200, 300], maximum tree depth [5, 10, 20, 30], minimum samples required for splitting [2, 5, 10], and minimum samples as are necessary for leaf nodes [1, 2, 4]. The best model was used for small molecule screening.

### 2.5. Homology Modeling of BCL2

We generated a homology model of BCL2 using SWISS-MODEL [39] since all available experimental crystal structures of BCL2 have unmodelled residues in the RCSB PDB database (https://www.rcsb.org/, accessed on 22 March 2024). The target sequence was downloaded from UniProt (https://www.uniprot.org/uniprotkb/P10415/entry#sequences, accessed on 22 March 2024), and the crystal structure with the PDB ID 5JSN was selected as the template. The resolution and the Rfree value of the template were 2.10 Å and 0.205, respectively. The best model containing the residues from Ser22 to Arg142 was used as the target in the subsequent molecular docking simulations.

### 2.6. AutoDock Vina (Vina) Molecular Docking

We prepared the ligands for docking using Open Babel [40] and GNU Parallel [41] implemented in POAP [42]. We used a docking protocol reported previously [43]. The coordinates for the center of the search space were −14.226, 1.146, and −10.800 along the x-, y-, and z-axis, respectively, for a cubic grid box with 26 Å sides. These parameters ensured site-specific docking for the Venetoclax binding site of BCL2 3D (PDB ID: 6O0K). We used the default grid spacing of 0.375 Å and the default exhaustiveness value of eight for docking simulations using Vina implemented in POAP.

### 2.7. Molecular Dynamics (MD) Simulations

We utilized the ‘Solution Builder’ module of CHARMM-GUI [44] to generate inputs for MD simulations using Gromacs (version 2023.2) [45]. We adopted a simulation protocol reported previously [43] Briefly, the simulation cell was energy-minimized with the steepest descent algorithm. The lincs constraint algorithm was implemented, and the system was equilibrated for 100 ps at 300 K using the V-rescale thermostat. This step was followed by an NPT (constant number of particles, pressure, and temperature) ensemble at 1 bar and 300 K, employing the C-rescale barostat and the V-rescale thermostat for an additional 100 ps. Finally, the constraints were removed, and the production MD runs were performed using the same NPT ensemble.

We then analyzed the MD simulation trajectories for root-mean-square deviation (RMSD), root-mean-square fluctuation (RMSF), solvent-accessible surface area (SASA, radius of gyration (Rg), and hydrogen bonds. Hydrogen occupancy was calculated using VMD [46]. 

## 3. Results

### 3.1. Activity Dataset

**Figure 1:**
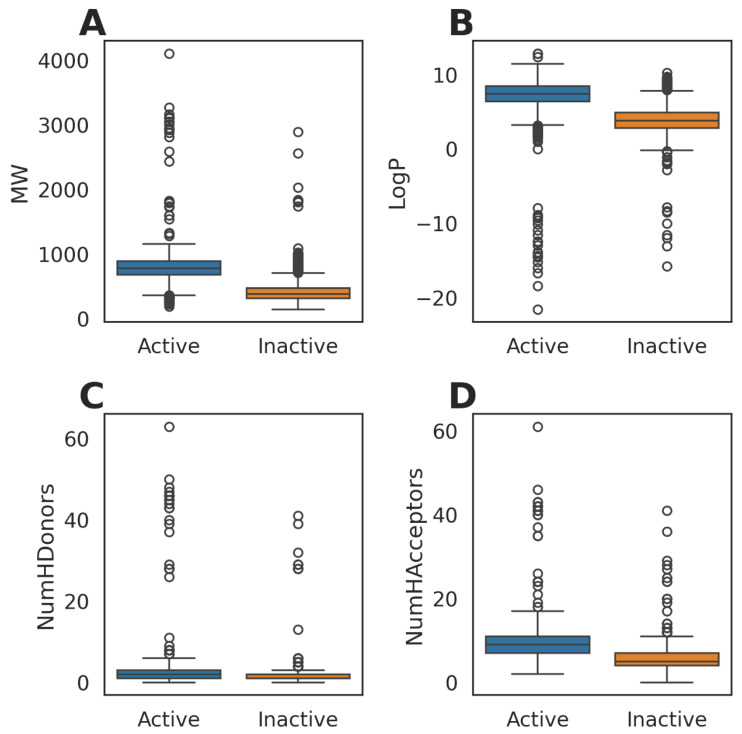
Lipinski descriptors of compounds tested in confirmatory bioassays for BCL2 activity. (**A**) Molecular weight (MW), (**B**) LogP, (**C**) number of hydrogen bond donors (NumHDonors), and (**D**) number of hydrogen bond acceptors (NumHAcceptors).

We retrieved the bioactivity data from PubChem, the world’s largest public database of 116 million chemicals [33], for BCL2 (PubChem gene id ‘596’). We filtered the records for ‘Confirmatory’ assays with a single target (BCL2) (Supplementary Data S1, retrieved on 13 October 2023). Next, we collected chemical information and activity records corresponding to 234 unique assay IDs and computed the Lipinski descriptors for 3867 compounds (Figure 1, Supplementary Data S2). 

Among 3867 compounds with activity records, 2631 were labeled as ‘Active’, and the remaining were treated as ‘Inactive’. The mean (±std) molecular weights for the ‘Active’ and ‘Inactive’ compounds were 781.98 (±246.47) and 428.41 (±202.84), respectively. Mean LogP values were higher for the ‘Active’ compounds (7.22 ± 2.43 vs. 3.88 ± 2.13). Compared to the ‘Inactive’ compounds, ‘Active’ compounds also had higher numbers of hydrogen bond donors (2.35 ± 3.80 vs. 1.56 ± 2.68) and hydrogen bond acceptors (9.08 ± 3.87 vs. 5.60 ± 3.14). 

At this point, we randomly split the data stratified for ‘Activity’ to use 95% of the records (3673 compounds) (Supplementary Data S3) for training and validating machine learning models and the remaining 5% of the records (194 compounds) (Supplementary Data S4) for external validation. 

### 3.2. Ligand Library for Screening

We implemented a comprehensive ligand-based virtual screening for BCL2 inhibitors. First, we saved data for 15 ligands (Table 1), which were complex in the BCL2 crystal structures in the RCSB PDB database. The inhibitors’ molecular weights ranged from 501.66 to 981.13, with four ligands having molecular weights of 500–600 and the remaining 11 ligands having 600–1000. 

Then, we extracted compounds from three databases, ChEMBL, DrugBank, and ZINC15, for each ligand, using SwissSimilarity [35] (Bragina et al., 2022), which created the ligand database of 7992 unique small molecules (Supplementary Data S5) that we screened using machine learning tools.

### 3.3. Deep Neural Network (DNN) Machine Learning Models

For the activity dataset of 3673 compounds (Supplementary Data S3), we generated the PubChem fingerprints from SMILES using PaDEL-Descriptor [34]. In total, 882 fingerprint features were generated for each compound (Supplementary Data S6). We used this activity dataset to develop a DNN predictive model designed to be flexible and customizable. The target variable was ‘Activity’, and the PubChem fingerprints were treated as features. Our grid search returned the best parameters: two sequential hidden layers with 256 and 128 neurons and 50 epochs with a batch size of 64.

The model architecture dealt with 1,035,859 total parameters (all trainable): 777,042, 225,792, 32,896, and 129 parameters for the first to the last layer, respectively. After validation, the best model (Supplementary Data S7) was returned with a test loss of 0.383 and a test accuracy of 0.959. The code is available in Supplementary Data S8.

**Figure 2:**
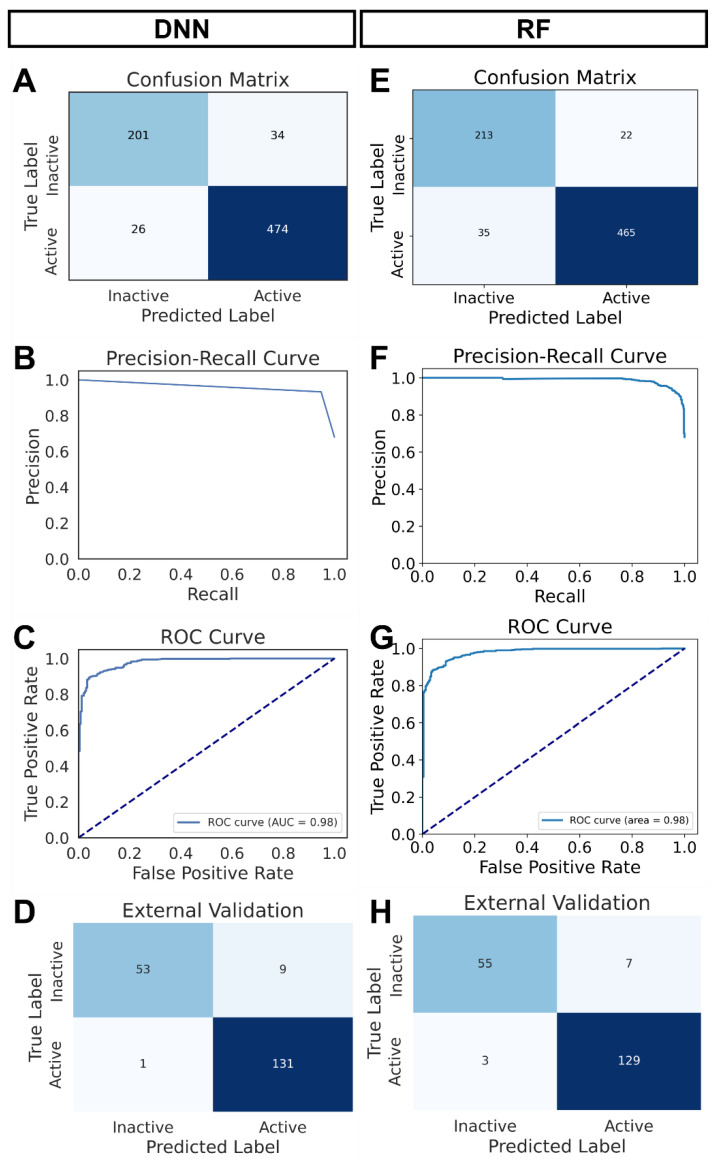
Performance matrix of the two machine learning models. (**A**–**D**) DNN model: confusion matrix (internal validation) (**A**), precision–recall curve (**B**), ROC curve (**C**), and confusion matrix (external validation) (**D**). (**E**–**H**) RF model: confusion matrix (internal validation) (**E**), precision–recall curve (**F**), ROC curve (**G**), confusion matrix (external validation) (**H**).

The performance metrics of the DNN model are provided in Figure 2. The confusion matrix (Figure 2A) reveals that the true-positive rate and the true-negative rate were 94.8% (474/(474 + 26)) and 85.5% (201/(201 + 34)), respectively. An AUC of 0.98 was found in the receiver operating characteristic (ROC). For the external validation dataset (Supplementary Data S4), the DNN model exhibited a true-positive rate of 99.2% and a true-negative rate of 85.5% (Figure 2D). Overall, the DNN model showed a high discriminatory power to distinguish small molecules as ‘Active’ or ‘Inactive’ for BCL2 inhibition (Figure 2A–D). 

### 3.4. Random Forest (RF) Machine Learning Model

Next, we generated an RF binary classification model with the same dataset we used for the DNN model (Supplementary Data S3). An initial grid search was used to identify the best parameters for building the RF model. We used 100 trees in the RF ensemble, and the maximum depth of each tree was set to 30. Each leaf node contained at least two samples, and a minimum of 10 samples was required to split an internal node. The model was then fitted to the training data and subsequently evaluated with the test data. The performance matrices of the RF model are given in Figure 2.

For the 235 instances of the ‘Inactive’ class, 213 were labeled correctly, and 22 were misclassified. In contrast, out of 500 active molecules, 465 were true positives, and 35 were false negatives. The precision was 86% for the ‘Inactive’ class and 95% for the ‘Active’ class. On the other hand, the recall (sensitivity) was 91% for the ‘Inactive’ class and 93% for the ‘Active’ class. Thus, the model performed very well classifying ‘Inactive’ and ‘Active’ molecules targeting the human BCL2 protein. Nevertheless, the performance was slightly better in predicting true ‘Active’ than the true ‘Inactive’. The ROC curve indicates an AUC of 0.98, suggesting the RF model’s overall high discriminatory power (Figure 2E–H).

We also evaluated the performance of the RF model using the external validation data (Supplementary Data S4). Of 62 true inactive molecules, 55 were correctly predicted as ‘Inactive’ (Figure 2H). Again, among 132 true active molecules, 129 were predicted correctly as ‘Active’. The precision and recall for the ‘Active’ class were 98% and 95%, respectively, indicating that the RF model can reliably predict true inhibitors for BCL2. The RF model was saved (Supplementary Data S9), and the corresponding code is provided in Supplementary Data S10.

The matrices revealed equal overall performance when we compared the DNN and the RF model (Figure 2). Considering the simplicity and lower computational resource requirement, we decided to use the RF model for screening the small molecule inhibitors for BCL2. 

### 3.5. Screening Small Molecules Using the RF Model

We estimated the Lipinski descriptors of the small molecules to be screened. The Lipinski’s rule of five (Ro5) is widely used to assess the likelihood of a compound’s success as an orally active drug [47]. The criteria are as follows: molecular weight (MW) should be less than or equal to 500 Daltons, calculated LogP (partition coefficient) should be less than or equal to 5, number of hydrogen bond donors (NumHDonors) should be less than or equal to 5, and number of hydrogen bond acceptors (NumHAcceptors) should be less than or equal to 10. If a compound has less than two violations, it is labeled as ‘Yes’, indicating that it is likely to possess drug-like properties.

**Figure 3:**
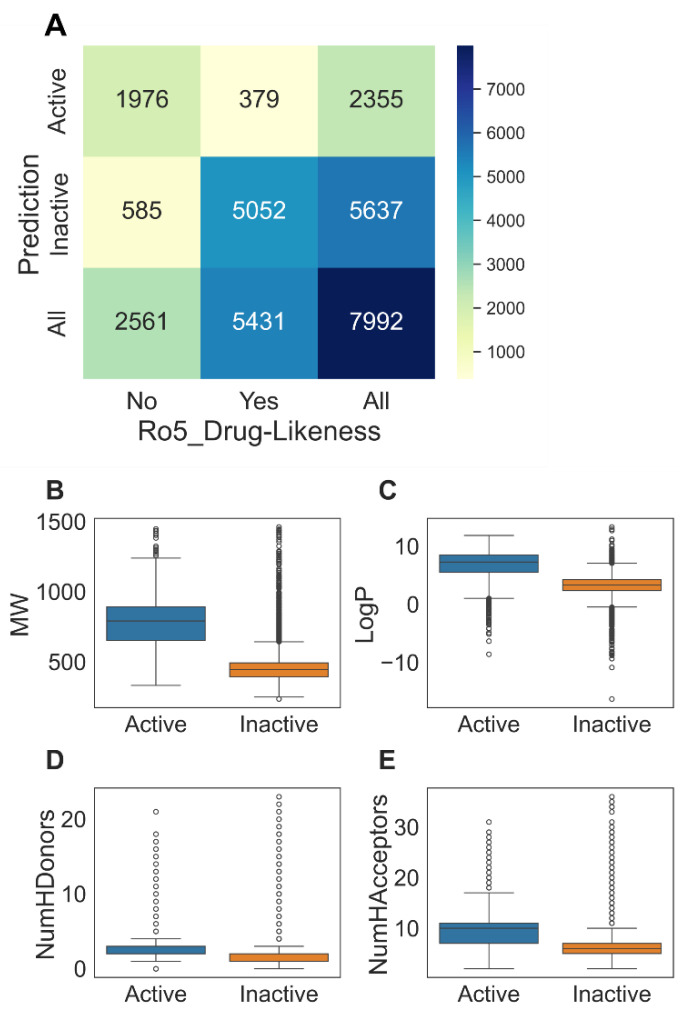
Summary of screening BCL2 inhibitors using the RF model. (**A**) A crosstab showing prediction and Ro5 druglikeness. (**B**) Molecular weight (MW), (**C**) LogP, (**D**) number of hydrogen bond donors (NumHDonors), and (**E**) number of hydrogen bond acceptors (NumHAcceptors) distribution among predicted ‘Active’ and ‘Inactive’ classes.

Next, we predicted the BCL2 inhibition activity of the small molecules using the RF model. The prediction results are summarized in Figure 3 and Supplementary Data S11. Among 7992 candidate molecules, the RF classified 2355 molecules as ‘Active’. Interestingly, only 16% (379/2355) of the ‘Active’ compounds passed the Ro5 criteria for druglikeness (Figure 3A). Overall, the ‘Active’ compounds exhibited higher MW, LogP, NumHDonors, and NumHAcceptors than the ‘Inactive’ compounds (Figure 3B–E).

### 3.6. Vina Molecular Docking

For further screening of the BCL2 inhibitors, we performed molecular docking using Vina. 

Table 2 lists the top ten hits based on the Vina docking scores. Scores for all docked compounds are available in Supplementary Data S12. Docking scores ranged from −5.8 kcal/mol to −11 kcal/mol. The score for Venetoclax, an FDA-approved drug and a known BCL2 inhibitor, was −9.8.

**Figure 4:**
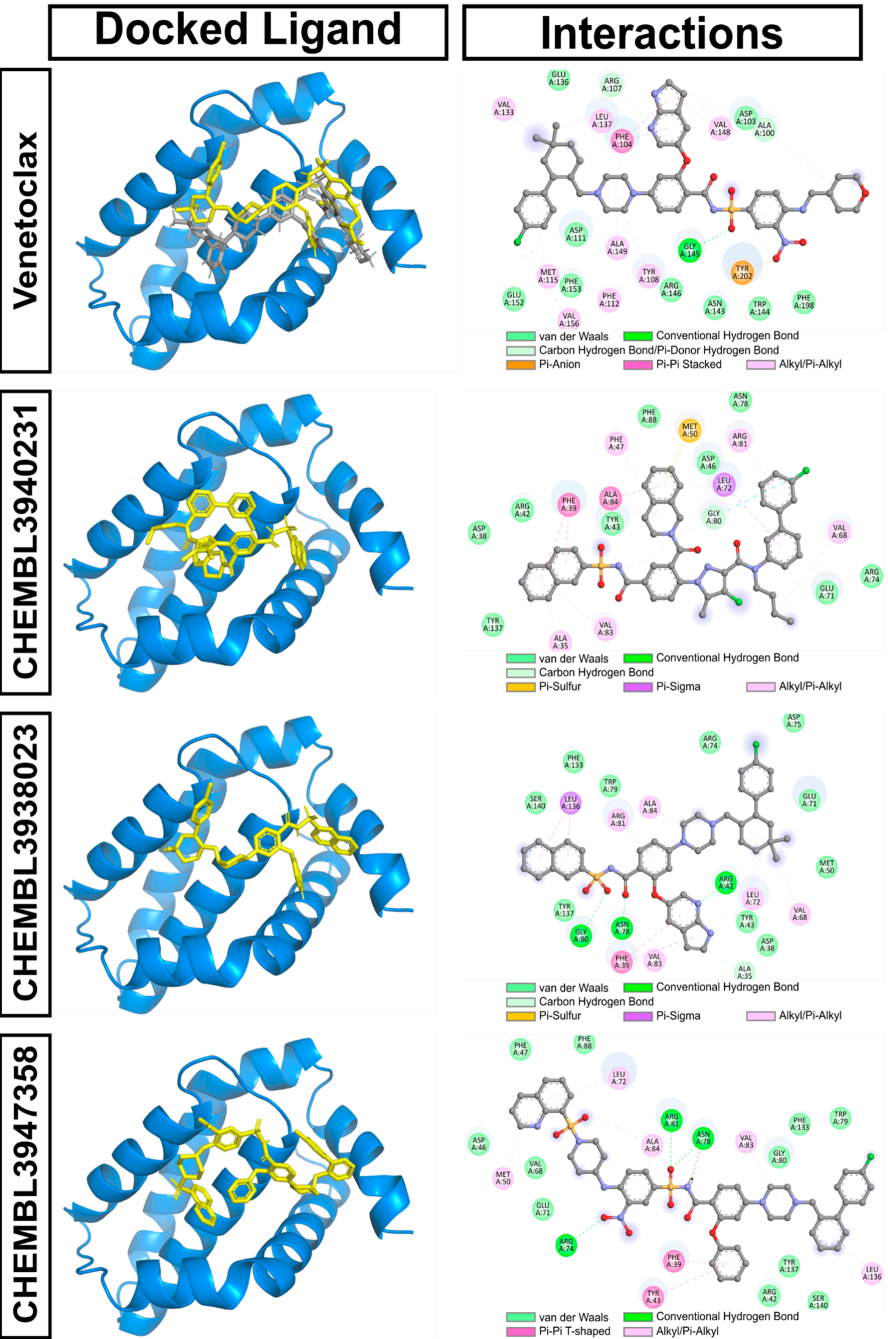
Docking pose and protein–ligand interactions of Venetoclax and the top hits. For the poses of docked ligands, the protein is represented by blue cartoons and the ligand by yellow sticks.

Figure 4 reveals the docking pose and protein–ligand interactions of Venetoclax and the top three hits. The binding poses of the redocked Venetoclax and the co-crystallized Venetoclax are apparently identical. Two-dimensional protein–ligand interaction analysis indicated that Venetoclax forms a conventional hydrogen bond with Gly145 and hydrophobic interactions with many BCL2 residues. On the other hand, CHEMBL3940231 is predicted to form mostly hydrophobic interactions. Interestingly, CHEMBL3938023 can interact with BCL2, forming hydrogen bonds with Arg42, Asn78, and Gly80. Additionally, this ligand interacts with multiple hydrophobic interactions. Similarly, CHEMBL3947358 shows hydrogen bonds with Arg74, Asn78, and Arg81 and many hydrophobic interactions (Figure 4).

### 3.7. Molecular Docking Simulations

**Figure 5:**
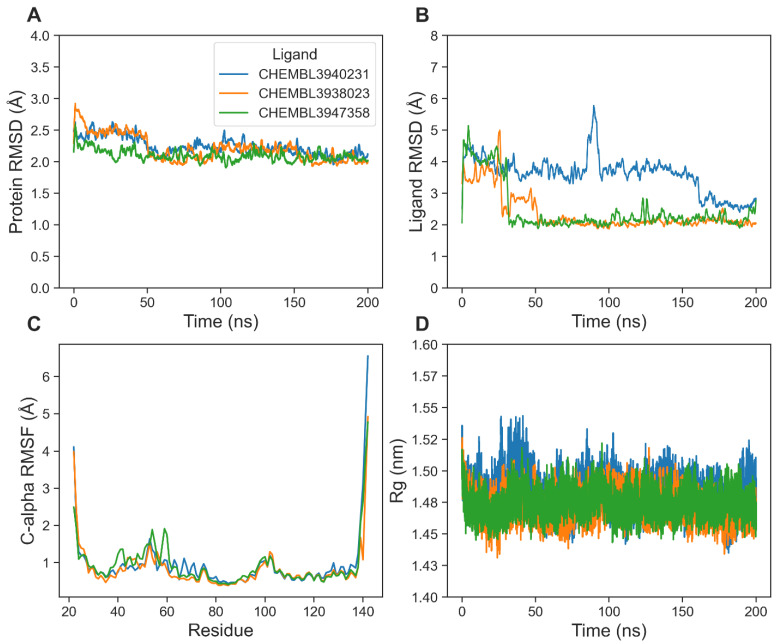
Global structural fluctuations. (**A**) Protein backbone RMSD. (**B**) Ligand (fitted to protein backbone) RMSD. (**C**) Per residue C-alpha RMSF. (**D**) Protein Rg.

To validate the results of our virtual screening using machine learning and docking, we performed molecular dynamics simulations for the complexes of the top three ligands. We first computed root-mean-square deviation (RMSD), a widely used measure of protein and ligand stability in a complex. Figure 5A reveals that the overall RMSD values of the protein backbone were below 3 Å in all cases. RMSD values were stabilized within 50 ns after slight initial fluctuations (Figure 5A). The mean (±std) ligand RMSD values were 3.65 (±1.01) Å, 2.44 (±0.60) Å, and 2.88 (±0.96) Å for CHEMBL3940231, CHEMBL3938023, and CHEMBL3947358, respectively. The ligand CHEMBL3938023 exhibited the lowest mean with a minimum fluctuation. Both CHEMBL3938023 and CHEMBL3947358 showed convergence within 50 ns (Figure 5B). However, CHEMBL3940231 showed wide fluctuation. Overall, consistently low ligand RMSD values indicated that CHEMBL3938023 remained stable at the binding pocket (Figure 5B).

We next plotted per residue fluctuations, as measured by the RMSF values, of the protein C-alpha atoms (Figure 5C). We noticed a higher local fluctuation from the loop residues Leu54-Ala61 for CHEMBL3940231. Apart from this, there were no remarkable differences in RMSF values among the top complexes. We then analyzed the Rg (Figure 5D), a reliable indicator of protein folding. A higher Rg indicates an extended conformation, while a lower Rg denotes a condensed form. Consequently, a significant change in Rg may reveal protein instability. The mean (±std) Rg values were 1.48 (±0.02) Å, 1.47 (±0.01) Å, and 1.48 (±0.02) Å for CHEMBL3940231, CHEMBL3938023, and CHEMBL3947358, respectively. Therefore, the protein structures with all three ligands revealed overall stable protein folding without any significant expansion or condensation (Figure 5D). 

**Figure 6:**
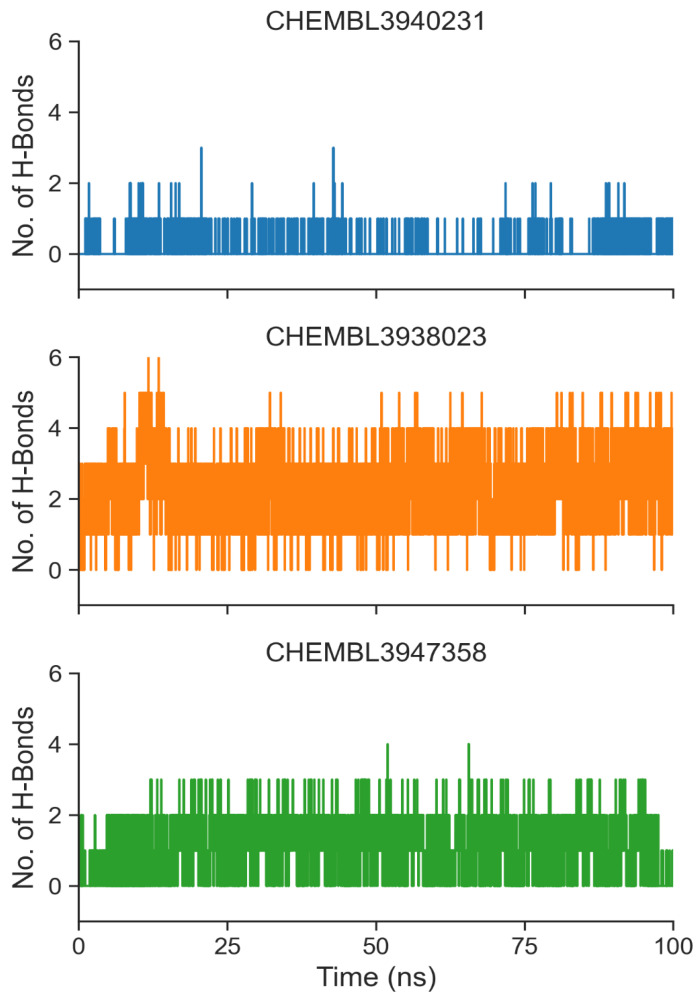
Formation of hydrogen bonds over time for the top ligands.

Hydrogen bonds play critical roles in protein–ligand binding. We calculated numbers on hydrogen bonds between the ligand and BCL2 from the simulation trajectories (Figure 6). Our analysis revealed that all three ligands could form at least one hydrogen bond during the 200 ns simulations. However, the highest number of bonds was found for CHEMBL3938023. Moderate interactions were apparent for CHEMBL3947358, while the lowest hydrogen bonds were formed for CHEMBL3940231 (Figure 6). We also calculated hydrogen bond occupancies of the top ligands (Table 3). Like the results depicted in Figure 6, many hydrogen bond donor–acceptor pairs were revealed for CHEMBL3938023. Notably, a stable hydrogen bond was established between CHEMBL3938023 and Arg42, exhibiting a 32.87% occupancy, and with Asp46 showing a 14.89% occupancy (Table 3). CHEMBL3947358 also established hydrogen bonds with Asn78 and Tyr43 with 6.99% and 23.53% occupancy, respectively (Table 3). On the contrary, there was no stable hydrogen bond donor–acceptor pair for CHEMBL3940231.

## 4. Discussion

Integrating machine learning with molecular docking can significantly improve the performance of virtual screening protocols. Singh et al. utilized eight machine learning models, including tree bagged forest, RF, Bayesian support vector machine, logistic regression, neural network, and neural network with bagging [48]. These models were trained using derivatized solvent accessible surface area (SASA) descriptors and showed enhanced performance compared to traditional scoring functions like Surflex and GOLD. Notably, neural networks and random forest models demonstrated superior virtual screening results, with up to a seven-fold increase in enrichment factors at 1% of the screened collections, making these techniques particularly effective for identifying hit compounds in protein–protein interaction datasets. 

Wen et al. conducted a study to identify new BCL2 inhibitors using a QSAR-based virtual screening approach [49]. They utilized random forest classification and regression models for screening the SPECS database, which led to the identification of compound M1 as a potential BCL2 inhibitor. This compound downregulates Bcl-2 expression significantly, induces mitochondrial dysfunction, and exhibits notable anti-cancer effects in breast cancer cells. The study demonstrated that compound M1 significantly reduced cell proliferation and clonogenicity in a dose-dependent manner while also proving harmful for known pain assay interference (PAINS) substructures.

In this study, we developed a method combining random forest algorithms, molecular docking, and molecular dynamics simulations to screen small molecules that could potentially inhibit BCL2, a known target in cancer therapy. The virtual screening process effectively narrowed down a large library of compounds, from which we identified top candidates based on their Vina docking scores, ranging from −5.8 kcal/mol to −11 kcal/mol. Notably, CHEMBL3940231 emerged as the top-scoring compound with a docking score of −11 kcal/mol, followed closely by other promising candidates like CHEMBL3938023 and CHEMBL3947358. CHEMBL3938023 demonstrated the ability to form hydrogen bonds with crucial BCL2 residues. MD simulations further supported this, where CHEMBL3938023 showed the lowest mean ligand RMSD, indicating a stable interaction within the BCL2 binding pocket.

Lipinski’s rule of five descriptors’ results indicate that, on average, active compounds have a higher molecular weight and lipophilicity than inactive compounds. Moreover, active compounds tend to have more hydrogen bond donors and acceptors. This distinction in physicochemical properties between active and inactive compounds aligns with the understanding that active compounds often possess certain structural features that enable them to interact effectively with their biological targets, in this case, BCL2 [50]. Statistically significant differences (*p* < 0.001) in these descriptors between active and inactive compounds underscore the potential of these properties in predicting the activity of new compounds. 

Our ligand library of 7992 unique small molecules, constructed by extracting compounds from three major databases—ChEMBL, DrugBank, and ZINC15—presents a diverse range of molecular weights (500–1000 Da), indicating a variety of structural frameworks and potentially different modes of interaction with the BCL2 protein. This range ensures that both lighter compounds, which often have better absorption and distribution profiles, and heavier compounds, which may engage more robustly with larger protein targets, are included in the screening process [51]. In addition, Lipinski’s rule of five states that the MW should not be more than 500. However, many approved drugs (including the BCL2 inhibitor Venetoclax) have a higher MW. Therefore, drug screening using Lipinski’s Ro5 is likely to miss true hits [52]. Therefore, our study included candidate molecules of relatively diverse sizes. This ensures a thorough exploration of the chemical space pertinent to the BCL2 target, thereby broadening the spectrum of potential therapeutic candidates. 

The precision–recall curve results demonstrated that the DNN model reached a precision of 93% and a recall of 95% for the active class, indicating a high rate of correct predictions but with a slightly higher chance of false positives than the RF model. The RF model achieved a precision of 95% for the active class, indicating a high likelihood of correct predictions when it labels a compound as active. The decision to utilize the RF model for subsequent screening processes stemmed from its equal performance in classification accuracy, simplicity, and more efficient use of computational resources compared to the DNN model. Furthermore, the RF model’s inherent interpretability, which is critical in the drug discovery process for understanding feature importance, favored its selection [53]. The RF model’s advantages in processing efficiency and lower computational demands make it particularly suitable for large-scale virtual screening tasks required in the study.

The ChEMBL records indicate that the top three chemicals, CHEMBL3940231, CHEMBL3938023, and CHEMBL3947358, are being used in preclinical research and have not been approved as drugs. All three share some similarities with the known BCL2 inhibitor, Venetoclax. For example, they are relatively large molecules with molecular weights between 700–1000, and all have four types of heteroatoms: chlorine, sulfur, nitrogen, and oxygen. Among Venetoclax and the top three compounds, CHEMBL3947358 is the largest, with the highest number of hydrogen bond acceptors. Both Venetoclax and CHEMBL3947358 have a nitro (-NO2) group, which is absent in CHEMBL3940231 and CHEMBL3938023. CHEMBL3940231 has a butyl side chain, while Venetoclax and CHEMBL3938023 have two methyl groups. Therefore, the top ligands share many physicochemical properties similar to the potent inhibitor, while the differences may indicate novel modes of interactions with the target (https://www.ebi.ac.uk/chembl/, accessed on 21 April 2024).

Overall, this study not only highlights the utility of integrating machine learning with molecular docking and MD simulations in drug discovery but also paves the way for developing novel BCL2 inhibitors that could contribute significantly to treating diseases where BCL2 plays a pivotal role. Our study only implemented computational methods. However, future laboratory experiments are required to validate the results of our study due to the predictive limitations of computational biology methods in drug discovery. Indeed, given that BCL2 is a prime target for cancer treatment, the top compounds identified in this study could be validated through various cell culture experiments, such as cell viability assays, caspase activation tests, mRNA expression profiling, and other validation tests, to assess their anticancer potential.

## 5. Conclusions

In conclusion, this study presents a method for the virtual screening of small molecules targeting BCL2, utilizing RF algorithms, molecular docking, and MD simulations. Identifying promising candidates such as CHEMBL3938023 and their subsequent analyses through docking poses and MD simulations suggests their potential as BCL2 inhibitors. 

## Figures and Tables

**Table 1:** Ligands complexed in the BCL2 crystal structures.

PDB ID	Ligand ID	Ligand Formula	Ligand MW	Ligand SMILES
4LVT	1XJ	C47 H55 Cl F3 N5 O6 S3	974.613	CC1(CCC(=C(C1)CN2CCN(CC2)c3ccc(cc3)C(=O)NS(=O)(=O)c4ccc(c(c4)S(=O)(=O)C(F)(F)F)NC(CCN5CCOCC5)CSc6ccccc6)c7ccc(cc7)Cl)C
4LXD	1XV	C34 H38 Cl N5 O7 S	696.213	c1cc(ccc1C2=C(COCC2)CN3CCN(CC3)c4ccc(cc4)C(=O)NS(=O)(=O)c5ccc(c(c5)[N+](=O)[O-])NC6CCOCC6)Cl
4MAN	1Y1	C48 H52 Cl N7 O8 S	922.487	CN(C)CCOc1cccc(c1CN2CCN(CC2)c3ccc(c(c3)Oc4ccc5c(c4)cc[nH]5)C(=O)NS(=O)(=O)c6ccc(c(c6)[N+](=O)[O-])NCC7CCOCC7)c8ccc(cc8)Cl
4AQ3	398	C40 H41 Cl I N5 O5 S	866.21	CCCCN(CCCC)C(=O)c1c(c(n(n1)c2ccc(cc2C(=O)N3CCc4ccccc4C3)C(=O)NS(=O)(=O)c5ccc6ccc(cc6c5)I)C)Cl
1YSW	43B	C36 H30 N4 O5 S3	694.842	c1ccc(cc1)CCc2nc3cc(ccc3s2)c4ccc(cc4)C(=O)NS(=O)(=O)c5ccc(c(c5)[N+](=O)[O-])NCCSc6ccccc6
2W3L	DRO	C34 H30 Cl N5 O2	576.087	Cc1c(c(nn1c2ccccc2C(=O)N3Cc4ccccc4CC3CN)C(=O)N(c5ccccc5)c6ccccc6)Cl
6GL8	F3Q	C43 H42 N4 O6	710.817	c1ccc(cc1)N(c2ccc(cc2)O)C(=O)c3cc(n4c3CCCC4)c5cc6c(cc5C(=O)N7Cc8ccccc8CC7CN9CCOCC9)OCO6
6QGG	J1H	C44 H48 Cl N6 O7 S2	872.471	C[N+](C)(CCC(CSc1ccccc1)Nc2ccc(cc2[N+](=O)[O-])S(=O)(=O)NC(=O)c3ccc(cc3)N4CCN(CC4)Cc5ccccc5c6ccc(cc6)Cl)CC(=O)O
6QGK	J1Q	C30 H39 N5 O2	501.663	CCCCN(CCCC)C(=O)c1cc(n(n1)c2ccccc2C(=O)N3Cc4ccccc4CC3CN)C
6QGJ	J1T	C48 H51 F3 N4 O9 S3	981.13	COc1cc2c(cc1C(CN3CCCC3)c4ccc(cc4)c5ccc(cc5)C(=O)NS(=O)(=O)c6ccc(c(c6)S(=O)(=O)C(F)(F)F)NC(CCN7CCOCC7)CSc8ccccc8)OCO2
6O0K	LBM	C45 H50 Cl N7 O7 S	868.439	CC1(CCC(=C(C1)c2ccc(cc2)Cl)CN3CCN(CC3)c4ccc(c(c4)Oc5cc6cc[nH]c6nc5)C(=O)NS(=O)(=O)c7ccc(c(c7)[N+](=O)[O-])NCC8CCOCC8)C
2O2F	LI0	C36 H40 N4 O6 S2	688.856	CC(C)(CSc1ccccc1)Nc2ccc(cc2[N+](=O)[O-])S(=O)(=O)NC(=O)c3ccc(cc3)N4CCC(CC4)(Cc5ccccc5)OC
2O22	LIU	C30 H36 N4 O5 S2	596.761	CC1(CCN(CC1)c2ccc(cc2)C(=O)NS(=O)(=O)c3ccc(c(c3)[N+](=O)[O-])NC(C)(C)CSc4ccccc4)C
8U27	ULL	C26 H24 Br N3 O3	506.391	CC(C)OC(=O)Nc1ccc(cc1)C2=NN(C(C2)c3ccccc3)C(=O)c4ccc(cc4)Br
7LHB	XZD	C46 H53 Cl N7 O11 P S	978.445	CC1(CCC(=C(C1)c2ccc(cc2)Cl)CN3CCN(CC3)c4ccc(c(c4)Oc5cc-6ccnc6n(c5)COP(=O)(O)O)C(=O)NS(=O)(=O)c7ccc(c(c7)[N+](=O)[O-])NCC8CCOCC8)C

**Table 2:** Vina docking scores of top ten hits and Venetoclax.

ID	Docking Score	SMILES	MW
CHEMBL3940231	−11	CCCCN(C(=O)C1=NN(C(C)=C1Cl)C1=CC=C(C=C1C(=O)N1CCC2=CC=CC=C2C1)C(=O)NS(=O)(=O)C1=CC2=CC=CC=C2C=C1)C1=CC=CC(=C1)C1=CC=CC(Cl)=C1	870.86
CHEMBL3938023	−10.9	CC1(C)CCC(CN2CCN(CC2)C2=CC=C(C(=O)NS(=O)(=O)C3=CC4=CC=CC=C4C=C3)C(OC3=CN=C4NC=CC4=C3)=C2)=C(C1)C1=CC=C(Cl)C=C1	760.36
CHEMBL3947358	−10.8	[O-][N+](=O)C1=CC(=CC=C1NC1CCN(CC1)S(=O)(=O)C1=CC=CC2=CC=CN=C12)S(=O)(=O)NC(=O)C1=CC=C(C=C1OC1=CC=CC=C1)N1CCN(CC2=CC=CC=C2C2=CC=C(Cl)C=C2)CC1	972.55
CHEMBL3983989	−10.6	CC1(C)CCC(CN2CCN(CC2)C2=CC=C(C(=O)NS(=O)(=O)C3=CN=C(OCC4(F)CCOCC4)C(=C3)C(F)(F)F)C(OC3=CN=C4NC=CC4=C3)=C2)=C(C1)C1=CC=C(Cl)C=C1	911.42
CHEMBL2031007	−10.6	CCCN(CCC)C1=NC(=CC=N1)C1=CC=C(C=C1C(=O)N1CCC2=C(C1)C=CC=C2)C(=O)NS(=O)(=O)C1=CC2=CC=CC=C2C=C1	647.80
CHEMBL3958123	−10.6	CC1(C)CCC(CN2CCN(CC2)C2=CC=C(C(=O)NS(=O)(=O)C3=CC=C(NC4CCN(CC4)C4CC5=CC=CC=C5C4)C(=C3)[N+]([O-])=O)C(OC3=CC=C4NC=CC4=C3)=C2)=C(C1)C1=CC=C(Cl)C=C1	968.62
CHEMBL3654087	−10.5	CC1(C)CCC(CN2CCN(CC2)C2=CC=C(C(=O)NS(=O)(=O)C3=CC=C(N[C@H]4CCCN(C4)C4CCOCC4)C(=C3)[N+]([O-])=O)C(OC3=C(F)C=C4NC=CC4=C3)=C2)=C(C1)C1=CC=C(Cl)C=C1	954.57
CHEMBL2431929	−10.5	OC(=O)C1=C(O)C=C(C=C1)N(CC1=CC=C(C=C1)C1CCCCC1)C(=O)CN(CC1=CC=CC=C1C#N)S(=O)(=O)C1=C(F)C(F)=C(F)C(F)=C1F	727.71
DB03063	−10.5	COC1=CC(=CC(OC)=C1OCC1=CC=CC=C1)C(=O)N[C@@H](CC1=CC=CC=C1)[C@@H](O)CN(CC[C@@H]1CCC2OCOC2C1)C(=O)CCN1C(=O)C2=C(C=CC=C2)C1=O	805.92
CHEMBL2030847	−10.4	CCCCC1=C(C(CO)=NN1C1=CC=CC=C1)C1=C(C=C(C=C1)C(=O)NS(=O)(=O)C1=CC2=CC=CC=C2C=C1)C(=O)N1CCC2=C(C1)C=CC=C2	698.84
CHEMBL3137309(Venetoclax)	−9.8	CC1(C)CCC(CN2CCN(CC2)C2=CC=C(C(=O)NS(=O)(=O)C3=CC=C(NCC4CCOCC4)C(=C3)[N+]([O-])=O)C(OC3=CN=C4NC=CC4=C3)=C2)=C(C1)C1=CC=C(Cl)C=C1	868.45

**Table 3:** Hydrogen bond occupancy of top ligands.

Ligand	Donor	Acceptor	Occupancy
CHEMBL3940231	ASN78-Side-ND2	LIG143-Side-O4	0.15%
	ASN78-Side-ND2	LIG143-Side-O5	0.20%
	TYR43-Side-OH	LIG143-Side-O3	0.10%
CHEMBL3938023	LIG143-Side-N4	ASP38-Side-OD1	2.40%
	ASN78-Side-ND2	LIG143-Side-O2	5.09%
	ARG42-Side-NH1	LIG143-Side-N3	32.87%
	LIG143-Side-N2	ASP46-Side-OD2	8.09%
	TYR43-Side-OH	LIG143-Side-O1	1.00%
	LIG143-Side-N2	ASP46-Side-OD1	14.89%
	LIG143-Side-N4	ASP38-Side-OD2	3.75%
	ASN78-Side-ND2	LIG143-Side-O3	0.95%
	ARG42-Side-NH2	LIG143-Side-N3	0.75%
	ARG42-Side-NE	LIG143-Side-N3	0.05%
	LIG143-Side-C30	ASP38-Side-OD1	0.05%
	LIG143-Side-N4	ARG142-Side-OT2	0.25%
	LIG143-Side-N4	ARG142-Side-OT1	0.15%
CHEMBL3947358	ASN78-Side-ND2	LIG143-Side-O8	0.05%
	ASN78-Side-ND2	LIG143-Side-O2	2.50%
	ARG81-Side-NE	LIG143-Side-O1	0.05%
	ASN78-Side-ND2	LIG143-Side-O1	0.20%
	ASN78-Side-ND2	LIG143-Side-O3	6.99%
	TYR43-Side-OH	LIG143-Side-O1	23.53%
	ASN78-Side-ND2	LIG143-Side-C47	0.05%
	TYR43-Side-OH	LIG143-Side-O4	0.05%
	TYR43-Side-OH	LIG143-Side-O5	0.10%

## Data Availability

Additional data is available upon request. Please email the corresponding author.

## Supplementary Materials

The following supporting information can be downloaded at: https://www.mdpi.com/article/10.3390/biom14050544/s1, Data S1: Pubchem Geneid 596 Bioassay; DataS2: BCL2 PubChem Activity Lipinski; Data S3: Activity Data; Data S4: External Validation Data; Data S5: All PDB SwissSimilarity; Data S6: Input Combined; Data S7: BCL2_DNN; Data S8: BCL2 DNN Analysis; Data S9: BCL2 RF Analysis; Data S10: RF Revision Analysis; Data S11: RF Screening Prediction SwissSimilarity; Data S12: Vina Docking Scores.
